# LncRNA IGBP1-AS1/miR-24-1/ZIC3 loop regulates the proliferation and invasion ability in breast cancer

**DOI:** 10.1186/s12935-020-01214-x

**Published:** 2020-05-07

**Authors:** Deqin Chen, Yangfan Fan, Fang Wan

**Affiliations:** Department of Surgery, The Women’s Hospital, School of Medicine, Zhejiang University, Hangzhou, Zhejiang China

**Keywords:** Breast cancer, IGBP1-AS1, miR-24-1, ZIC3

## Abstract

**Background:**

Breast cancer (BC) is one of the malignant solid tumors with the highest morbidity in the world. Currently, the therapeutic outcome of different types of treatment can be unsatisfactory. Novel lncRNA biomarkers in BC remains to be further explored.

**Methods:**

Different expression of lncRNAs among BC tissues and adjacent normal tissues were identified with microarray analyses. A series of in vivo and in vitro gain-of-function laboratory procedures were conducted to study the biological functions of IGBP1-AS1. The prognostic effects on IGBP1-AS1 survival were evaluated by using in situ hybridization and survival analysis. In addition, other experiments including RNA pull down analysis, RNA immunoprecipitation, luciferase reporter assays, and chromatin immunoprecipitation as well as validating assays conducted in vivo were applied to identify the target and regulatory mechanisms of IGBP1-AS1.

**Results:**

Significant down-regulation of IGBP1-AS1 was discovered in the cell lines and tissues of BC. With respect to its biological function, overexpression of IGBP1-AS1 had inhibitory effects on the invasion and proliferation of BC cells in vivo as well as in vitro. Analysis of the samples obtained from BC patients indicated a positive effect of IGBP1-AS1 on survival outcomes. LncRNA IGBP1-AS1/miR-24-1/ZIC3 axis as a loop can regulate the proliferation and invasion of BC cells.

**Conclusions:**

IGBP1-AS1 could have inhibitory impact on the invasion and proliferation of BC and may serve as a promising biomarker for BC.

## Background

The morbidity and mortality rates of breast cancer (BC) worldwide are more than 20% and 10%, respectively [1]. Currently, treatment for BC includes surgery, chemotherapy, radiotherapy, endocrine therapy and targeted therapy however, the therapeutic effect often remains unsatisfactory. Long-term continuous chemoradiotherapy can lead to toxic side effects and weaken the patient, while the cytotoxic effect on cancer cells is also poor [2, 3]. Current targeted therapies for BC mainly include targeted endocrine therapy for hormone receptor-positive BC and epidermal growth factor receptor (HER)-2-positive BC. However, the use of trastuzumab in the clinical treatment of early HER2-positive and metastatic BCs benefits less than 35% of patients [4], and the development of drug resistance is an additional problem that needs to be urgently solved.

Human genomics research has often focused on RNAs that encode proteins. RNAs that cannot encode proteins are considered useless RNAs or transcriptional noise. However, these RNAs play an indispensable role in important activities such as cell proliferation, differentiation, senescence and apoptosis. In mammalian genomics, only a small number of transcripts encode proteins, and the vast majority are noncoding RNAs (ncRNAs), accounting for approximately 80% of the human genome. According to the size of the transcript, ncRNAs can be divided into miRNAs and lncRNAs. NcRNAs greater than 200 nt in length are called lncRNAs [5]. Ponting et al. [6] studied the evolution of lncRNAs and their role in transcriptional regulation and epigenetic gene regulation. They speculated that lncRNAs may have five different origins: generated by (1) protein-coding gene sequence disruption; (2) chromatin rearrangement; (3) noncoding RNA reverse transcriptional translocation; (4) noncoding genes containing adjacent repeats; and (5) insertion of a transposable element into the genome. However, there is still no definitive theory about the origin of lncRNAs. The biological functions of lncRNA are mainly accomplished through signaling molecules, decoy molecules, guide molecules, scaffold molecules and chromatin modification complexes, occurring at both the transcriptional level and the posttranscriptional level, and by structural RNAs in cell proliferation, differentiation, senescence, death, carcinogenesis, and the occurrence and development of disease [7].

The expression of lncRNAs in BC tissues varies considerably. Different types of lncRNAs affect BC by interfering with mRNA splicing and inducing apoptosis, X chromosome silencing and genomic imprinting [8]. Genomics research provides a new feasible solution for the detection, diagnosis, target selection and prognostication of BC. In this study, differentially expressed lncRNAs in BC and para-carcinoma tissues were screened by gene chip technology; from these analyses, the lncRNA IGBP1-AS1 was identified, and its function was elucidated to reveal its clinical value. IGBP1-AS1 has not been studied before and therefore, its biological function as well as regulatory mechanism remained unclear. Furthermore, in this research we also confirmed the downstream targets of IGBP1-AS1. We found that miR-24-1 may be sponged by IGBP1-AS1. miR-24-1 has been reported to be involved in tumorigenesis or chemoprevention of colorectal cancer, ovarian cancer, pediatric pilocytic astrocytomas and ependymomas [9–11]. Zic family member 3 (ZIC3) is a transcription factor that has widely studied. Its function varies in several cancers [12, 13], however, in BC, it still remains unknown.

## Methods

### Microarray profiling

The LncRNA microarray expression profiling was carried out strictly based on the protocol provided by the manufacturer to screen the LncRNAs with varied expression levels in BC tissues and adjacent tissues (fold change > 1.5 and *Padj*< 0.05). At first the target cDNA was synthesized, labeled and purified, after which the Cyanine-3-CTP labeled cRNA was hybridized with the lncRNA microarray chip. The samples were analyzed by microarray after washing. R project was applied for result analysis and clustering.

### Culture of cell lines

The BC cell lines (HCC70 and UACC-812) and human mammary cells (76 N-F2V) used in this study were gained from American Type Culture Collection (ATCC, Manassas, VA, USA). These cells were incubated in RPMI-1640 medium with 10% fetal bovine serum (FBS) and a supplement of 100 μg/L streptomycin and 100 μg/L penicillin under the conditions of 37 °C and 5% CO_2_.

### Real-time quantitative polymerase chain reaction (RT-qPCR)

The TRIzol reagent (Invitrogen, USA) was applied for total RNA extraction, and the SYBR Green Mix (Promega) was adopted for primer amplification (forward primer: AGCAGCATTTTCCTGGCTAC; reverse primer: GGTGGAGGGGGAACCCATAG). The synthesis work of the primers was provided by Zhejiang East Genecreate Biological Engineering Co., Ltd. Data analysis was conducted by using the2^−ΔΔCt^ method. The miR-24-1 expression was determined with the TaqMan MicroRNA Assays Kit (Applied Biosystems, USA). U6 and GAPDH were adopted as the endogenous controls for miR-24-1 and IGBP1-AS1. All the experiment procedures were conducted in triplicate.

### Cell transfection with lentivirus

The pBLLV-CMV-IRES-ZsGreen IGBP1-AS1, pCMV-IRES-GFP ZIC3 cDNA lentiviral plasmid for overexpression (oe) transfection and Phblv-u6-ZsGreen-Puro ZIC3 shRNA lentiviral plasmid for knock-down transfection were gained from Genelily BioTech Co., Ltd, (Shanghai, China). The transfection procedure was performed with Lipofectamine 3000 (Invitrogen). The transfected cells were cultured with puromycin (2 μg/mL) for 2 weeks, and the stable cells were selected. The RT-qPCR technique was adopted for transfection efficiency verification.

### Cell Counting Kit-8 (CCK8) assay

After a 5-day cell incubation at 37 °C and 5% CO_2_, CCK8 solution was added to each well of the 96-well plates (2 × 10^3^ cells/well). A microplate reader was applied to detect the cell viability by measuring the absorbance value at 450 nm.

### MTT assay

After 24, 48, 72 and 96-h cell incubation, 10 μL MMT (5 mg/mL) was added to each well of the 96-well plate (1 × 10^4^ cells/well), and the transfected BC cells were cultured for another 4 h. After removal of the upper layer, 100 μL DMSO was added. The absorbance value at 490 nm was detected with a microplate reader.

### Flow cytometry

A previous developed protocol was applied for the flow cytometry assay [14]. Cell apoptosis was analyzed with the Annexin V-FITC early apoptosis kit, and the flow cytometer (FACScan; BD Biosciences) was adopted to analyze the IGBP1-AS1overexpressed BC cells and negative control cells, the results of which was calculated by CellQuest software (BD Biosciences).

### Transwell assay

Transwell chambers (8-μm pore size; Corning Costar, USA) was used for the evaluation of cell invasion and migration. With the cells inoculated on the upper chamber, 20% serum was used as chemoattractant in the lower chamber. After 48-h incubation, methanol was used for fixation, and 0.1% crystal violet was applied for staining. Due to the slight abrasion on the upper surface of the filter, cells located on the lower surface were counted and photographed by using a microscope. All the procedures were carried out in triplicate.

### Wound-healing assay

An equivalent amount of HCC70 and UACC-812 cells that was transfected with IGBP1-AS1 over-expressed plasmid and negative control (NC) plasmid were inoculated into 6-well plates. After the cells adhered to the wall and formed monolayers, a gap was drawn in the middle of the cell layer with a pipette tip. After 24 h, a microscope was used to observe the BC cells that migrated into the gap area.

### Samples from tissue

The BC tissues and adjacent normal breast tissues used in this study were gained from the surgeries carried out in the Second Affiliated Hospital of Jiaxing University from Jan 2011 to June 2017 and stored at − 80 °C. Informed consents and complete clinical information was obtained from all the patients. In the end, microarray profiling was conducted with 10 BC specimens and 6 normal breast specimens (6 paired). The survival analysis and immunohistochemical assays were conducted in the other 94 BC specimens. The clinical procedures related to human were approved by the institutional human experiment and ethics committee of the Second Affiliated Hospital of Jiaxing University and the Affiliated Women’s Hospital of Zhejiang University. All the surgical procedures were carried out strictly in line with the provisions of the Helsinki Declaration.

### In situ hybridization (ISH)

The ISH assay was conducted in accordance with a previously developed method [15]. After labeling with the digoxigenin antibody (Roche, 11,093,274, 1:1000), the locked nucleic acid probe with complementary sequences of IGBP1-AS1 (custom LNA detection probe, Exiqon) was synthesized. The staining intensity was reviewed by 2 independent pathologists who were blinded to the study design.

### Luciferase reporter assays

The pmirGLO-IGBP1-AS1-wt reporter vector was prepared by cloning the IGBP1-AS1 cDNA with miR-24-1 predictive binding site into the pmirGLO Dual-Luciferase miRNA Target Expression Vector (Promega). A similar procedure was carried out with the IGBP1-AS1 cDNA with point mutations of the miR-24-1 seed region binding site to form the pmirGLO-IGBP1-AS1-Mut reporter vector. Then the miR-24-1 and miR-NC were transfected with the vectors into the HEK-293FT cells by using Lipofectamine 3000 (Invitrogen).

The analyses on the websites UCSC (http://genome.ucsc.edu/) and JASPAR (http://jaspar.genereg.net/) indicated that the ZIC3 protein might bind to the DNA sites of IGBP1-AS1. The IGBP1-AS1 recombinant luciferase reporter vector with truncated or mutated binding sites was constructed and co-transfected into the HCC70 cells with the ZIC3 expression vector to verify the specific sites of ZIC3 protein binding to IGBP1-AS1 DNA. After 48 h of transfection, the luciferase reporter assay was then carried out with a luciferase assay kit (K801-200, Biovision, USA) following the manufacturer’s instruction. With renilla luciferase as an internal reference gene, the activation degree of the target reporter gene was compared based on the ratio of the relative luciferase unit (RLU) of firefly luciferase assay divided by the RLU of Renilla luciferase assay.

### RNA immunoprecipitation (RIP)

The trial was carried out strictly based on the protocol of EZMagna RIP Kit (Millipore) provided by the manufacturer. In brief, after cell lysis with the complete RNA immunoprecipitation (RIP) lysis buffer, extract of the BC cells was incubated together with anti-argonaute 2 (AGO2) or control anti-IgG antibody conjugated magnetic beads at 4 °C for 6 h. The purified RNA was evaluated with the RT-qPCR after removing the proteins of the beads.

### RNA pull-down assay

The 3′end biotinylated miR-24-1 (TGCCTACTGAGCTGATATCAGT) or miR-24-1-mut (TGCCTACTCAGCTGATATCAGT) (20 nmol/L) were transfected into the HCC70 cells. After 24 h of incubation with streptavidin-coated magnetic beads (Life Technologies), pull down assay was conducted in a biotin-coupled RNA complex. Finally, the IGBP1-AS1 abundance was calculated based on the RT-qPCR results.

### Western blot assay

The RIPA lysis buffer with PMSF was applied for the extraction of the total protein in tissues and cells, which were incubated in an ice bath for 30 min. The lysate was centrifuged for 10 min under the condition of 4 °C and 8000*g* to collect the supernatant. The SDS-PAGE gel electrophoresis was performed with all the samples, and the separated protein was transferred to PVDF membrane. 5% skimmed milk was used for membrane blocking. After 1 h room-temperature blocking, the membrane was incubated together with primary sheep anti-ZIC3 (1:1000, ab215063, Abcam, UK) and rabbit anti-GAPDH (1:2500, ab9485, Abcam, UK) at 4 °C overnight, followed by 1-h incubation with HRP-labeled secondary antibody. Afterwards, the membrane was rinsed with TBST, and the proteins were visualized by using the ECL Fluorescence Detection Kit (Cat. No. BB-3501, UK) and photographed by Bio-Rad Image Analysis System (BIO-RAD, USA). The software of Quantity One v4.6.2 was adopted for quantification. The protein expression levels were presented as the relative gray values of the interested protein band and the GAPDH protein band. All the procedures were performed in triplicate and the final results were presented as mean values.

### Chromatin immunoprecipitation (ChIP)

Formaldehyde was used for cell fixation for 10 min. The chromatin fragments were obtained by cell sonication, and the supernatant was collected after centrifugation. The negative control rabbit IgG (ab109489, 1:300, Abcam, Shanghai, China) and the target protein-specific antibody ZIC3 (sc-101201, 1:1000, Santa Cruz Biotechnology, Shanghai, China) were added to fully incubate supernatant at 4 °C overnight. The protein agarose/Sepharose were used for DNA–protein complex precipitation, which was centrifugated at 12,000*g* for 5 min, followed by discard of the supernatant. After washing off the non-specific complex, cross-linking was conducted at 65 °C overnight. The DNA fragment was extracted, purified and recovered by phenol/chloroform. The primer was designed to amplify which contains the site of ZIC3 binding to the IGBP1-AS1 DNA promoter (F: 5′-CTTCATGGTGCAGGGTGCTA-3′, R: 5′-TGCATGTGGTTGTGCTCAGA-3′). The amplified product was 775 bp long. A Distal primer (a primer that amplifies the sequence away from the IGBP1-AS1 DNA promoter region) was designed as a negative control for the site primer (F: 5′-AGCTCATTTCTCCCCTTGCC-3′, R: 5′-TCTCTACTCCCACCAGAGGC-3′) and the amplified product was 384 bp long. Using the recovered DNA fragment as an amplification template, site primers and Distal primers were added respectively to perform RT-qPCR to verify whether the site of IGBP1-AS1DNA was the binding site of transcription factor ZIC3.

### Animal trials

The BALB/c-nude mice aged 4 to 5 weeks were obtained from Shanghai SLAC Laboratory Animal Co., Ltd. All the laboratory procedures related to animals were approved by the institutional animal care and use committees of both institutions. The concentration of the stably infected cells was adjusted to 5 × 10^6^ cells/mL after being suspended in 50% Matrigel (BD Biosciences, Bedford, MA). 0.4 mL cell suspension (2 × 10^6^ cells) was administrated to the nude mice by means of subcutaneous injection at the left axilla area. The tumor size was examined every 5 days, and volume of the tumor was calculated with the formula of length × width^2^ × 0.5. All nude mice were euthanized, and the tumors were weighed 30 days later.

The mice were submitted toa right lateral flank incision after anesthesia to construct the abdominal metastasis model. 100 μL Hank’s balanced salt solution containing HCC70-Luc-vector and HCC70-Luc cells (5 × 10^6^, transfected with Lv-IGBP1-AS1, miR-24-1 mimic, Lv-IGBP1-AS1 + Si-ZIC3 or Lv-ZIC3 + miR-24-1 mimic) was administrated into the right abdominal cavity by injection. 4 weeks after the operation, the bioluminescence images were collected by the Interactive Video Information System (IVIS).

### Statistical methods

All the numerical measurements were presented as mean ± SD. The student’s t test and one-way ANOVA test were applied for the evaluation of group differences according to the data nature. The overall survival time was defined as the period between the diagnosis and death of any cause, and the progression-free time duration started from the beginning of treatment and ended when disease progression occurred. Log-rank test and Cox’s regression analysis were applied for univariate and multivariate survival analyses. *P* < 0.05 was considered as the standard for statistical significance. All the analyses were conducted with SPSS 22.0 and the imagines were drawn with GraphPad Prism 7.0.

## Results

### Significant down-regulation of the LncRNA IGBP1-AS1 in BC tissues

**Fig. 1:**
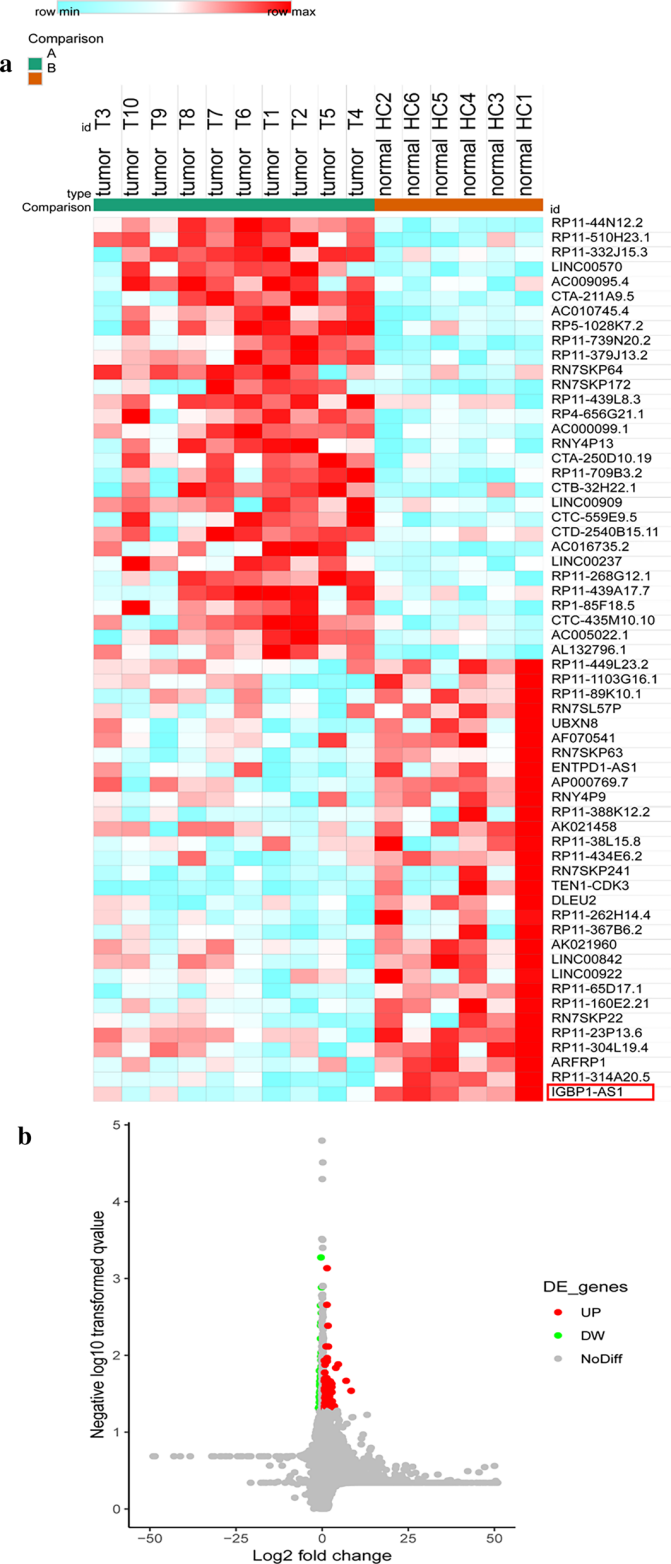
Down-regulation of the LncRNA IGBP1-AS1 in BC tissues was observed. **a** The heat map presented the 60 lncRNAs with differed expression levels in BC specimens and normal adjacent specimens (fold change > 1.5; *P *< 0.05). **b** The volcano plot of the LncRNAs with the colored dots representing the LncRNAs with Log_2_ (fold change) > 0.66 and *P *< 0.05

In total 60 lncRNAs with differed expression levels were identified (Fig. 1a, b). IGBP1-AS1 localized on chromosome Xq13.1 was significantly decreased in BC tissues (Fig. 1a)

### Inhibitory effect on viability and invasion of BC cells induced by IGBP1-AS1

**Fig. 2:**
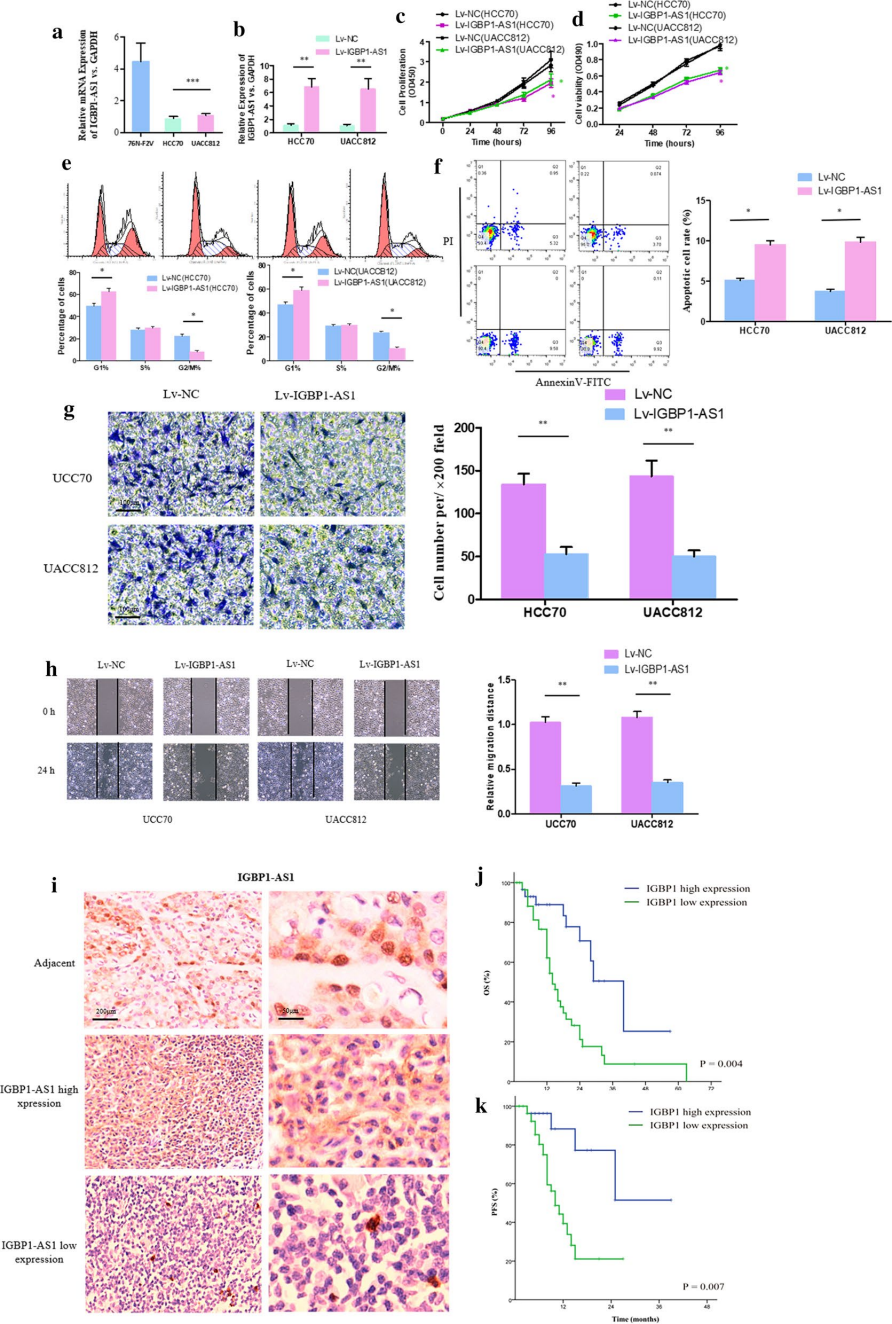
In-vitro inhibitory effect of the IGBP1-AS1 on BC cell proliferation. **a** The RT-qPCR indicated the expression of IGBP1-AS1 in BC cell lines (HCC70 and UACC-812) relative to human mammary cells (76N-F2V). **b** The RT-qPCR demonstrated up-regulated efficiency of IGBP1-AS1 in BC cells. **P value < 0.01. **c**, **d** Assessment of cell proliferation and viability by CCK8 and MTT assays. *P value < 0.01. **e** The FACS analysis indicated overexpression of IGBP1-AS1 may reduce the number of cells in the G2/M phase. *P value < 0.01. **f** Increased expression level of IGBP1-AS1 could promote the apoptosis of BC cells according to the Annexin V assay. **P value < 0.01. **g**, **h** The results of the transwell assay and wound-healing assay performed with HCC70 and UACC-812 cells transfected with Lv-IGBP1-AS1 or Lv-NC. **P value < 0.01 vs. control. **i** The expression level of IGBP1-AS1 in BC tissues and adjacent normal tissues according to the ISH assay. **j**, **k** Comparison of the Kaplan–Meier curves of the BC patients with respect to OS rate and PFS rate respectively

The RT-qPCR demonstrated that the expression level of IGBP1-AS1 in BC cell lines was significantly down-regulated compared to the normal human mammary cells (76 N-F2V) (*P *< 0.05) (Fig. 2a). The cell proliferation and viability (obtained through CCK8 and MTT assays) in the IGBP1-AS1 overexpressed group were reduced compared with the negative control group (Fig. 2c, d). Furthermore, the cell cycle was inhibited (Fig. 2e) and cell apoptosis was enhanced in the IGBP1-AS1 overexpressed group (Fig. 2f). The transwell assay and wound-healing assay indicated that the increased expression of IGBP1-AS1 could have significant inhibitory impact on the cell’s invasive capacity (*P *< 0.05) (Fig. 2g, h).

### Positive impact of IGBP1-AS1 on the survival outcomes of BC patients

There were 94 BC tissues with complete clinical information included in survival analysis. According to the ISH assay, the expression level of IGBP1-AS1 in BC tissues was lower compared to the adjacent normal tissues (Fig. 2i). The expression level of IGBP1-AS1 was classified based on the staining intensity score in ISH (score 0–2: low expression; score 3–4: high expression). Table 1 contains the patient characteristics at baseline. The median overall survival (OS) was 40 months in the high expression of IGBP1-AS1 group and 14 months in the low expression of IGBP1-AS1 group (Fig. 2j). The median progression-free survival (PFS) of the low expression group was 10 months, which was significantly longer than that of the high expression group (*P* value < 0.001) (Fig. 2k). The multivariate analyses verified that IGBP1-AS1 expression in BC could be an independent risk factor for the OS (HR: 3.102, 1.365 to 6.526, *P *= 0.005) (Table 2). Another independent factor is disease staged at II, III or IV. With respect to the PFS, lower IGBP1-AS1 expression level could also lead to worse survival outcome regarding disease progression (HR: 4.472, 1.565 to 13.926, *P *= 0.009) (Table 3). These results indicate that IGBP1-AS1 could have a positive effect on the survival outcomes in BC patients.

**Table 1:** Clinical characteristics of patients with breast cancer

Characteristic	All patients	IGBP1-AS1 High expression	IGBP1-AS1 Low expression	*P*-value
Total	94	30	64	
Age (years)				0.382
< 60	74	22	52	
≥ 60	20	8	12	
Menopause				
No	69	23	46	0.624
Yes	25	7	18	
Invasion depth				
T1	13	3	10	0.461
T2–4	81	27	54	
Lymph node metastasis				
N0	38	9	29	0.158
N1–3	56	21	35	
Stage				
I	30	7	23	0.222
II/III/IV	64	23	41	
Histologic grade				
I/II	58	20	38	0.498
III	36	10	26	
Histologic type				
Ductal	81	27	54	0.461
Lobular	13	3	10	
ER				
Negative	47	13	34	0.376
Positive	47	17	30	
PR				
Negative	63	21	42	0.674
Positive	31	9	22	
HER2				
Negative	46	12	34	0.235
Positive	48	18	30	

*ER* estrogen receptor, *PR* progesterone receptor, *HER2* human epidermal growth factor receptor 2

**Table 2:** Univariate and multivariate analysis of breast cancer patients on overall survival

Variable	Univariate analysis		Multivariate analysis	
	HR (95% CI)	*P*	HR (95% CI)	*P*
Age (≥ 60 vs. < 60 years)	1.033 (0.997–1.062)	0.057		
Menopause (yes vs. no)	1.107 (0.620–1.875)	0.134		
Stage (II/III/IV vs. I)	2.726 (1.256–4.789)	*0.021*	2.035 (1.477–3.329)	*0.011*
IGBP1-AS1 expression (low vs. high)	3.144 (1.494–6.616)	*0.003*	3.102 (1.365–6.526)	*0.005*
Histologic grade (III vs. I/II)	1.373 (0.384–2.513)	0.463		
Histologic type (lobular vs. ductal)	1.208 (0.507–3.391)	0.601		
ER (yes vs. no)	0.827 (0.384–2.731)	0.379		
PR (yes vs. no)	0.595 (0.277–1.982)	0.301		
HER2 (yes vs. no)	1.282 (0.893–2.191)	0.115		

Italic values indicate significance of *P* value (*P* < 0.05)

*ER* estrogen receptor, *PR* progesterone receptor, *HER2* human epidermal growth factor receptor 2

**Table 3:** Univariate and multivariate analysis of breast cancer patients on progress free survival

Variable	Univariate analysis		Multivariate analysis	
	HR (95% CI)	*P*	HR (95% CI)	*P*
Age (≥ 60 vs. < 60 years)	1.103 (0.908–1.462)	0.094		
Menopause (yes vs. no)	1.002 (0.521–1.745)	0.173		
Stage (II/III/IV vs. I)	1.957 (1.052–2.789)	*0.032*	1.713 (0.9524–2.329)	0.067
IGBP1-AS1 expression (low vs. high)	4.602 (1.559–13.588)	*0.006*	4.472 (1.565–13.926)	*0.009*
Histologic grade (III vs. I/II)	1.076 (0.448–2.273)	0.416		
Histologic type (lobular vs. ductal)	1.198 (0.407–3.131)	0.592		
ER (yes vs. no)	1.072 (0.568–1.591)	0.214		
PR (yes vs. no)	1.315 (0.650–2.081)	0.324		
HER2 (yes vs. no)	1.482 (0.749–2.438)	0.195		

Italic values indicate significance of *P* value (*P* < 0.05)

*ER* estrogen receptor, *PR* progesterone receptor, *HER2* human epidermal growth factor receptor

### Verification of impact of IGBP1-AS1 in vivo

**Fig. 3:**
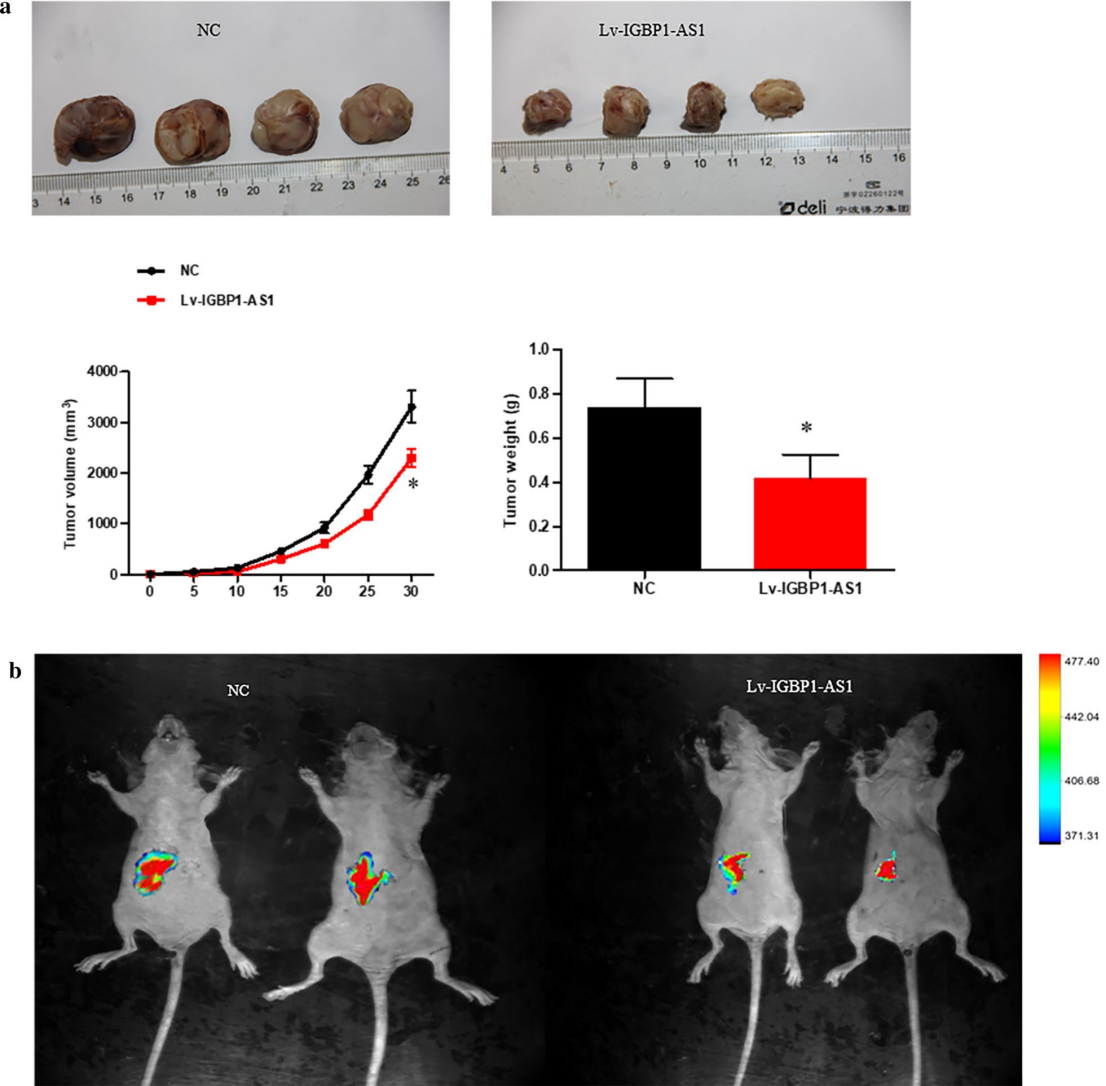
In-vivo effect of the IGBP1-AS1 on metastasis and proliferation. **a** Animal trial performed by means of a subcutaneous injection of the HCC70 cells. **b** The luciferase activity of the HCC70 cells and control cells collected by bioluminescent imaging. **P value < 0.01

In our study, we found that tumor tissues with highly expressed IGBP1-AS1tended to be smaller in volume and lower in weight (Fig. 3a). To validate the impact of IGBP1-AS1 on metastases, we performed an abdominal metastasis model. According to the bioluminescence imaging, overexpressed IGBP1-AS1 could significantly inhibit cell invasion in vivo (*P *< 0.05) (Fig. 3b).

### IGBP1-AS1 targeted and regulated miR-24-1

**Fig. 4:**
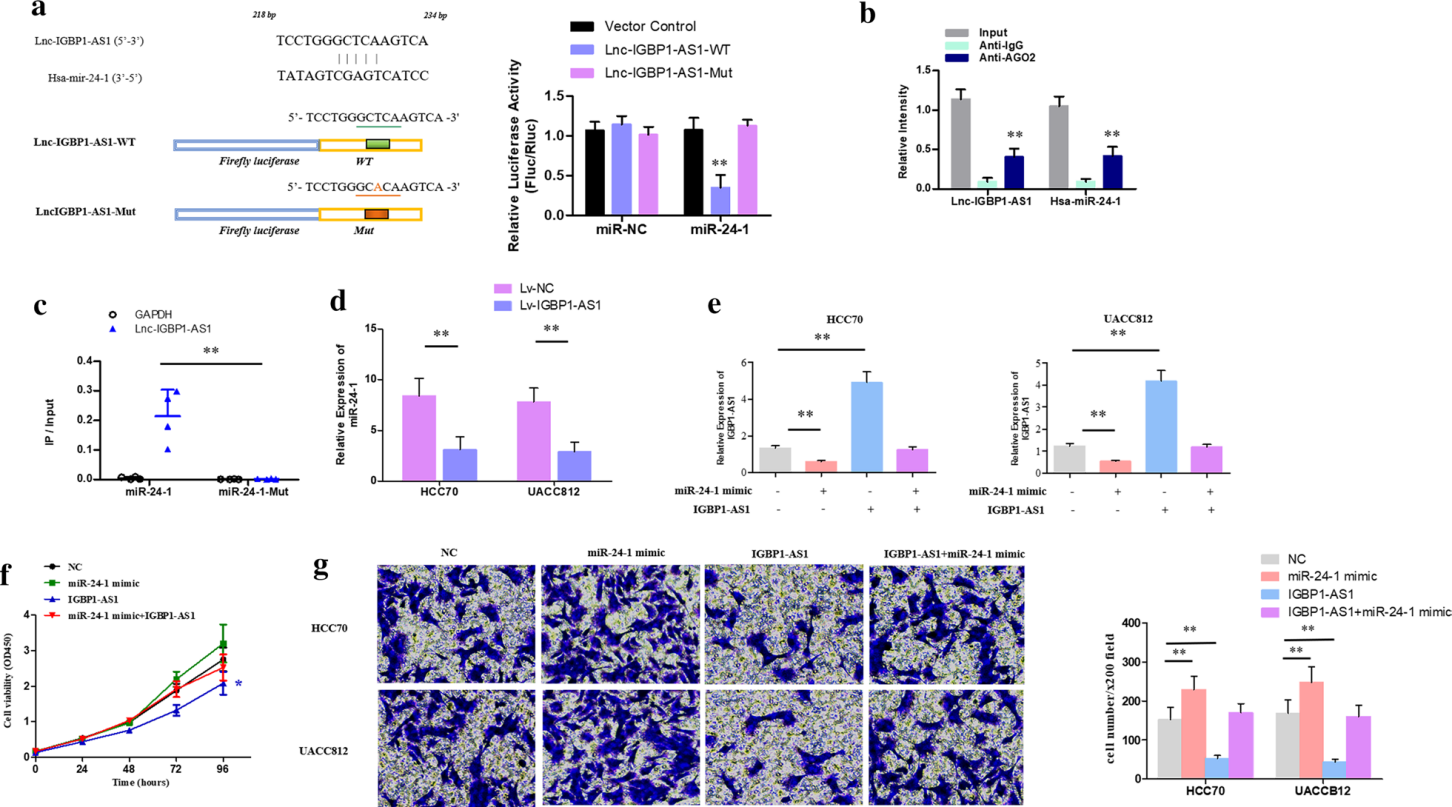
Regulatory effect of the IGBP1-AS1 on miR-24-1. **a** The predictive binding sites of IGBP1-AS1 with miR-24-1 was shown in the upper panel of the schematic diagram. The histogram presented the luciferase activities of the HCC70 cells. **P value < 0.01. **b** According to the anti-AGO2 RIP assays, the levels of IGBP1-AS1 and miR-24-1 were higher in anti-AGO2 compared to the anti-IgG immunoprecipitates. **P value< 0.01. **c** As shown in the scatter plot, the level of IGBP1-AS1 was higher in the miR-24-1-wt than in miR-24-1-mut. **P value < 0.01. **d** The expression level of miR-182 in BC cells with overexpressed IGBP1-AS1 relative to the cells with overexpressed-NC. The relative expression of IGBP1-AS1 (**e**) and cell proliferation (**f**) after transfection were assessed by the RT-qPCR and CCK-8 assay respectively. *P value < 0.05. **g** The BC cell invasion after transfection was evaluated with the transwell assay. *P value < 0.05

The target miRNA with reversed complementary sequence of IGBP1-AS1 was predicted by bioinformatics (miRcode http://www.mircode.org/), and miR-24-1 was selected for further research. Based on the results of the luciferase reporter assay of pmirGLO-IGBP1-AS1-wt reporter vector, the luciferase activity in the HCC70 cells transfected with miR-24-1 was significantly reduced compared to the cells transfected with miR-NC. With respect to the pmirGLO-IGBP1-AS1-mut reporter vector, the luciferase activity of the two groups did not differ from each other (Fig. 4a). The results of the RIP assay indicated that IGBP1-AS1 and miR-24-1 were particularly enriched in AGO2 compared to anti-IgG immunoprecipitates (Fig. 4b). In addition, the RNA pull-down assay suggested that the level of IGBP1-AS1 was higher in the miR-24-1-wt than in the miR-24-1-mut (Fig. 4c). Furthermore, the RT-qPCR demonstrated a decreased expression of miR-24-1 in BC cells with overexpressed IGBP1-AS1 (Fig. 4d). The weakened expression of IGBP1 (Fig. 4e) and the enhancement of cell proliferation (Fig. 4f) and invasion (Fig. 4g) caused by the miR-24-1 mimic could be largely reversed by overexpressed IGBP1-AS1 in the rescue experiments.

### In-vivo/in vitro modulation effect of the Lnc IGBP1-AS1/miR-24-1/ZIC3 axis on the invasion and proliferation of BC cells

**Fig. 5:**
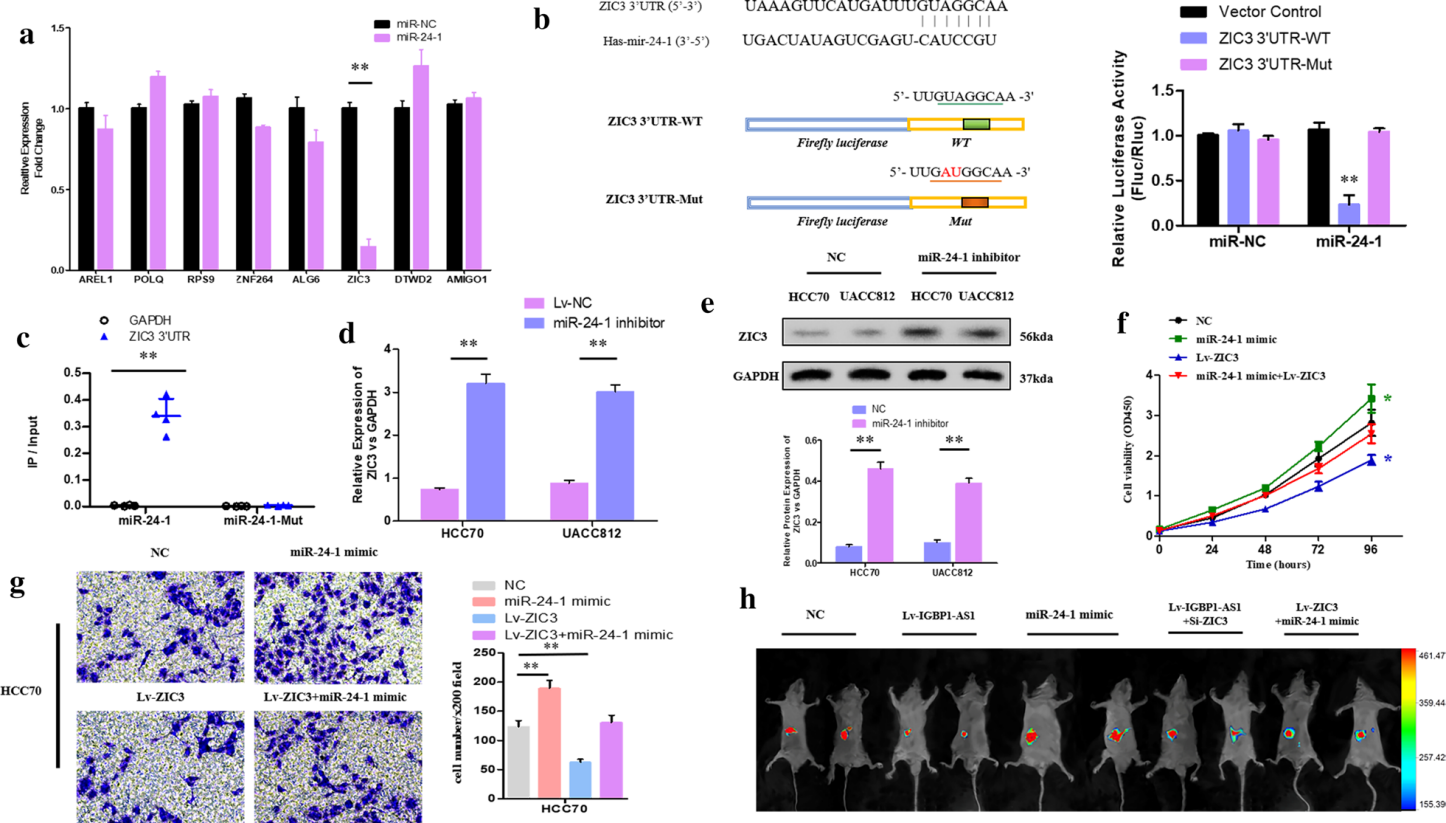
ZIC3 could be the target of miR-24-1. **a** The RNA quantification of the HCC70 cells transfected with miR-24-1 and miR-NC according to the RT-qPCR. **P value< 0.01. **b** The schematic diagram listed the conjectural miR-24-1 binding sites within the 3′UTR of ZIC3 as well as the sequences of wild-type and mutant 3′UTR of ZIC3. The luciferase activity in HCC70 cells was detected by using luciferase reporter gene assays (**P value < 0.01). **c** The scatter plot presented the relative RNA levels of the 3′UTR of ZIC3 and GAPDH that were quantified by the RT-qPCR after transfection in the HCC70 cells. (**P value< 0.01). **d**, **e** The expression level of mRNA and protein of ZIC3 in melanoma cells with miR-24-1 inhibitor and NC were presented. **f** Cell proliferation according to the CCK-8 assay (*P value< 0.05). **g** HCC70 cell invasive capacity based on the transwell assay (**P value < 0.01). The validation of IGBP1-AS1/miR-24-1/ZIC3 axis in vivo. **h** The bioluminescent imaging of the mice was collected by IVIS bioluminescence imaging system after transplantation of luciferase expressing HCC70 cells transfected with NC, Lv-IGBP1-AS1, miR-24-1 mimic, Lv-IGBP1-AS1 + Si-ZIC3, Lv-ZIC3 + miR-24-1 mimic

The binding sites in several genes’ 3′-UTR with miR-24-1 were predicted by bioinformatics (Targetscan 7.2; miRDB; miRTarBase). Zic Family Member 3 (ZIC3) was considered as the target gene based on the screening of RT-qPCR (Fig. 5a). The luciferase assay showed that the 3′-UTR of wild-type ZIC3 had the ability to significantly inhibit the luciferase activity of the cells transfected with miR-24-1, though it had no impact on the cells transfected with miR-NC. In addition, 3′-UTR of mutant-type ZIC3 did not significantly influence the luciferase activity of the cells transfected with miR-24-1 (Fig. 5b). It was confirmed in the RNA pull-down assay that the content of ZIC3 3′-UTR was higher in the wild-type miR-24-1 compared to the mutant-type miR-24-1 with mutant ZIC3 3′-UTR binding site (Fig. 5c). The western blot assay and RT-qPCR suggested that the level of miR-24-1 could have an impact on the mRNA and protein expression in ZIC3 (Fig. 5d, e). Moreover, the up-regulation of miR-24-1 in BC cells had reversal effect on the inhibition of cell viability (Fig. 5f), invasion and migration (Fig. 5g) induced by ZIC3 overexpression. The HCC70 cells that transfected with NC, Lv-IGBP1-AS1, miR-24-1 mimic, Lv-IGBP1-AS1 + Si-ZIC3 and miR-24-1 mimic + Lv-ZIC3 were administrated into the abdominal cavity of the BALB/c-nude mice by injection. The results observed in the abdominal metastasis model were similar to those in vitro (Fig. 5h).

### IGBP1-AS1 might be further up-regulated by recruiting its downstream target: transcription factor ZIC3

**Fig. 6:**
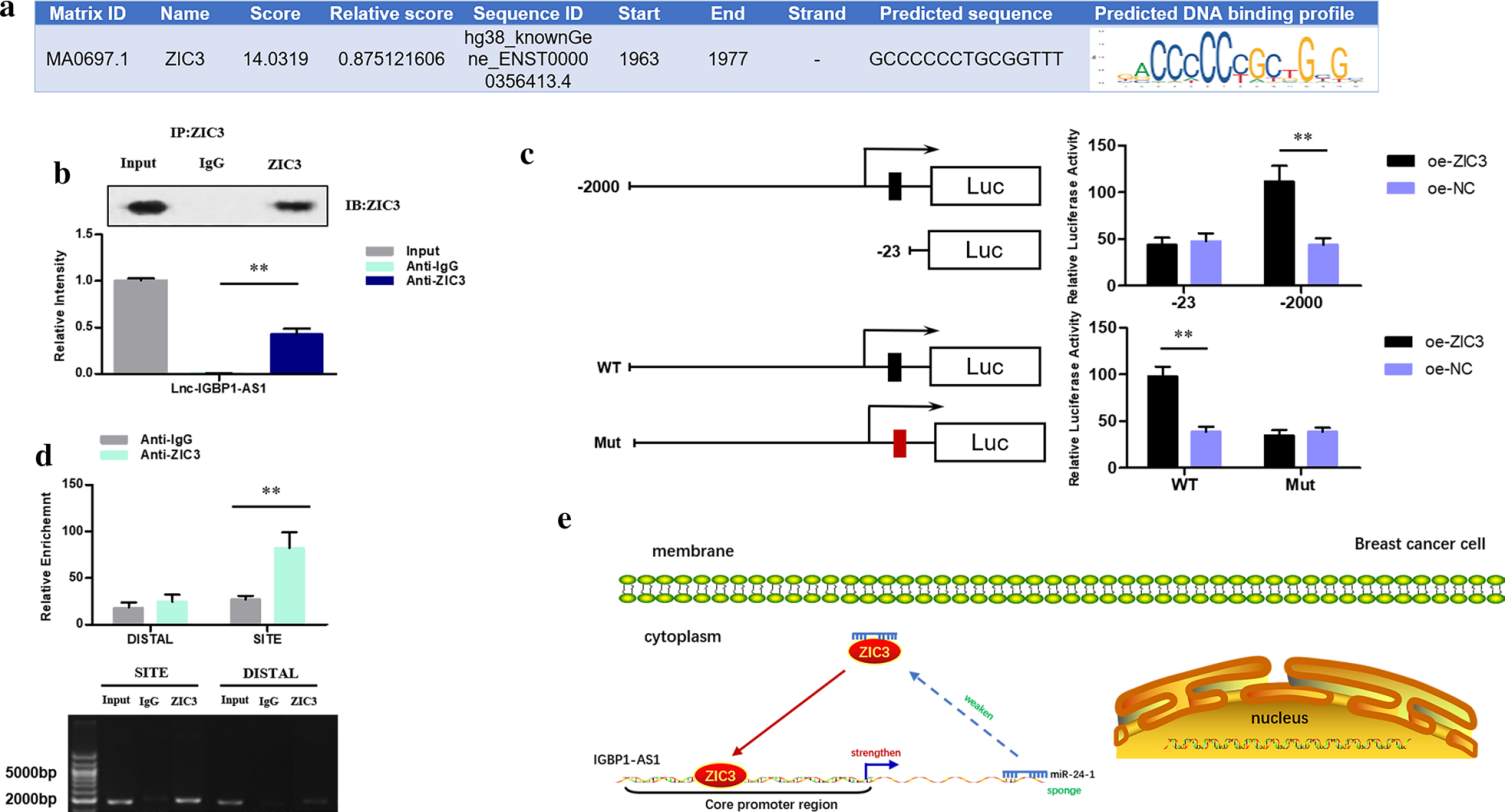
**a** Predicted site where ZIC3 protein may bind to IGBP1-AS1 DNA. **b** RIP assay to identify the binding of IGBP1-AS1 to ZIC3. **P value < 0.01. **c** Upper Panel: Dual luciferase reporter assay to identify fluorescence intensity in cells with co-transfected truncated IGBP1-AS1 recombinant luciferase reporter vector and ZIC3 expression vector. **P value < 0.01; Lower Panel: dual luciferase reporter assay to identify fluorescence intensity of the cells with co-transfected mutated IGBP1-AS1 recombinant luciferase reporter vector and ZIC3 expression vector. **P value < 0.01. **d** ChIP assay to identify the binding ability of ZIC3 to the IGBP1-AS1 DNA binding site. **P value < 0.01. **e** Schematic representation of the regulation of the IGBP1-AS1/miR-24-1/ZIC3 loop in BC. IGBP1-AS1 up-regulates the level of ZIC3 via suppressing miR-24-1. On the other hand, the elevated ZIC3 synchronously promotes the expression of IGBP1-AS1 by binding to its DNA at promoter region, further inhibiting BC cell proliferation and invasion

The target gene ZIC3 was well-known as a transcription factor. To determine whether it could bind to IGBP1-AS1 at the promoter region, we searched and predicted the most possible site of ZIC3 protein binding to IGBP1-AS1 DNA by UCSC (http://genome.ucsc.edu/) and JASPAR (http://jaspar.genereg.net/) (Fig. 6a). The RIP assay verified the binding of IGBP1-AS1 to ZIC3 (Fig. 6b). Compared with the IgG, the binding of ZIC3 to IGBP1-AS1 was notably increased (*P* < 0.05), and the anti-ZIC3 antibody could precipitate IGBP1-AS1, indicating that IGBP1-AS1 can form a complex with ZIC3. Furthermore, the luciferase reporter assay (Fig. 6c) demonstrated a significant up-regulation in the ability of oe-ZIC3 to activate IGBP1-AS1 (P < 0.05). When the site was truncated or mutated, luciferase activity was the same compared to the cells transfected with oe-NC. These results confirmed that the predicted site was indeed the ZIC3 protein binding to IGBP1-AS1 DNA site. Next, the binding ability of ZIC3 to the IGBP1-AS1 DNA binding site was validated by ChIP assay in HCC70 cells (Fig. 6d). The amount of amplification products obtained from site primers in the ZIC3 group was larger than from the Distal primers in IgG group, while there was no significant difference in the amount of amplification products between the two pairs of primers in the IgG group. The results indicated that the predicted site of IGBP1-AS1 DNA was indeed the binding site to transcription factor ZIC3. It could be deduced that when IGBP1-AS1 up-regulates the level of ZIC3 via suppressing miR-24-1, the elevated ZIC3 could synchronously promote the expression of IGBP1-AS1. Synergistic action might be observed.

## Discussion

Previously, studies have identified some BC-related lncRNAs. HOTAIR is a kind of lncRNA that is found in human fibroblasts and is transcribed in antisense, and its expression is abnormally elevated in breast cancer and its metastases. The expression of HOTAIR results in increased invasion of breast cancer cells, which is closely related to the progression of breast cancer [16], thus leading to decreased survival and poor prognosis. HOTAIR is also a prognostic indicator for breast cancer. Gokmen-Polar et al. [17] believed that its use for prognostication is only suitable for breast cancer patients with ER-negative disease and lymph node metastases. Therefore, the significance of HOTAIR as a prognostic indicator for breast cancer is relatively limited. Of course, there are different opinions. Milevskiy et al. [18] found that HOTAIR was co-expressed with FOXA1 and FOXM1 in HER2 receptor-rich tumors when analyzing breast cancer-related gene expression data. Li et al. [19] screened a group of lncRNA microarrays involving trastuzumab-resistant SKBR-3/Tr cells and found that the expression of GAS5 is decreased in breast cancer patients and SKBR-3/Tr cells after trastuzumab treatment, demonstrating that trastuzumab can reduce the expression of GAS5. It was found that lapatinib upregulated the expression of GAS5 by inhibiting the PI3K/Akt/mTOR signaling pathway. GAS5 acts as a competitive endogenous RNA (ceRNA) of miR-21, competitively binding to miR-21 to increase PTEN and promote proliferation and metastasis of trastuzumab-resistant breast cancer cells. This process improves the therapeutic resistance of breast cancer to trastuzumab. Finally, it has been shown that GAS5 can improve the therapeutic resistance of HER2-positive breast cancer to trastuzumab, suggesting that GAS5 can be used as a novel prognostic indicator and candidate drug target for HER2-positive breast cancer and can improve the effect of treatment on trastuzumab-resistant patients.

Our findings suggest that expression of the lncRNA IGBP1-AS1 was remarkably lower in BC tissues and cell lines than in adjacent normal tissues. The in vivo imaging system showed that BC cells overexpressing IGBP1-AS1 had less invasive ability in the peritoneal cavity of mice than the control cells, which was also confirmed in vitro. At the same time, a retrospective analysis was performed on tumor tissues from 94 patients with BC. Multivariate analysis showed that IGBP1-AS1 expression was an independent risk factor affecting both OS and PFS and was not affected by disease stage. The median OS and PFS in the IGBP1-AS1high expression group were remarkably higher than those in the IGBP1-AS1low expression group, indicating that IGBP1-AS1 has clinical value in the prognosis of BC. Loss-of-function assays were not performed in this study as the level of IGBP1-AS1 in BC were relatively low. Remarkable changes in phenotypes may not be observed when knock-down was conducted. The rescue experiments have proved that to some extent. Further mechanism research has identified that LncRNA IGBP1-AS1/miR-24-1/ZIC3 axis has significant impact on proliferation and invasion ability of BC cells in vitro and in vivo. miR-24-1 had never been reported in BC before. According to our results, it may act as an oncogene in BC, which was in accord with studies in colorectal cancer and ovarian cancer [9, 10]. It was not the first time that miR-24-1 was involved in loop-feedback regulation [20]. Some members of the ZIC family has been reported as tumor suppressor in gastric cancer including ZIC3 [12]. Interestingly, we found that ZIC3 also binds to IGBP1-AS1 at the promoter region making the axis a ring loop which is rare. It is a favorable feedback regulation in BC which may present another new therapeutic strategy. Although this regulation might be weakened or compensated in the human body. There were also some limitations. Sample size for survival analysis needs to be enlarged greatly to make bigger clinical impact as well as performing a prospective study. The signaling pathways that were affected by the LncRNA IGBP1-AS1/miR-24-1/ZIC3 loop need to be further explored.

## Conclusion

Expression of the LncRNA IGBP1-AS1 is decreased in BC, and in vitro and in vivo experiments confirm that its biological function in BC serves to resist tumor proliferation and invasion. The LncRNA IGBP1-AS1/miR-24-1/ZIC3 loop may be regarded as new therapeutic targets as it regulates proliferation and invasion of BC. However, the lncRNA IGBP1-AS1 is still rarely studied. An in-depth study of its biological functions and regulatory mechanisms could help us better understand the pathogenesis of BC, which could the main direction of future research.

## Data Availability

The datasets used and analyzed during the current study are available from the corresponding author on reasonable request.

## Abbreviations

ncRNAs: Noncoding RNAs
ATCC: American Type Culture Collection
FBS: Fetal bovine serum
RT-qPCR: Real-time quantitative polymerase chain reaction
ISH: In situ hybridization
DMSO: Dimethyl sulfoxide
RIP: RNA immunoprecipitation
AGO2: Argonaute 2
IVIS: Interactive Video Information System
